# Thank you to Reviewers and Editors 2023

**DOI:** 10.1093/plphys/kiae055

**Published:** 2024-02-05

Editor-in-Chief Yunde Zhao and the staff of *Plant Physiology* thank the reviewers listed below, whose insight and contributions in reviewing manuscripts from January 1 to December 31, 2023, have helped make the journal a success. We would also like to thank the Associate Editors, Monitoring Editors, and Assistant Features Editors for their contributions to the journal and recognize those Editors who have concluded their term of service, as well as those who joined the Editorial Board in 2023.

昊 王

Mostafa Aalifar

Fayezeh Aarabi

Mark G.M. Aarts

Moe Abbas

S.M. Abdul-Awal

Siti Nor Akmar Abdullah

Mitsutomo Abe

Steffen Abel

Maria Jazmin Abraham-Juarez

Cei Abreu-Goodger

Liana G. Acevedo-Siaca

V. Mohan Murali Achary

Natalia Patricia Achkar

Mingkee Achom

Guido Van den Ackerveken

Shunsuke Adachi

Zach Adam

Mattia Adamo

Kunal Adhikary

Charith Raj Adkar-Purushothama

Magaret Ahmad

Olivier Van Aken

Seiji Akimoto

Markus Albert

Nick W. Albert

Liza E. Alexander

Muhammad Ali

Andrew C. Allan

Douglas K. Allen

Randy D. Allen

Silke Allmann

Jose M. Alonso

Muhammad Ahsan Altaf

Burcu Alptekin

Marcio Alves-Ferreira

Jonas Chaves Alvim

Richard M. Amasino

Iraida Amaya

Charles Ampomah-Dwamena

Samuel Amsbury

Stig U. Andersen

Tonni Grube Andersen

Charles T. Anderson

Alexis De Angeli

Ruthie Angelovici

Koh Aoki

Takashi Aoyama

Pedro J. Aphalo

Giovanni Appendino

Elias Feitosa Araujo

Wagner L. Araújo

Agustín Lucas Arce

Federico D. Ariel

Steven A. Arisz

William Armstrong

Thomas Arnesen

Albrecht G. von Arnim

Laura Arribas-Hernández

Stéphanie Arrivault

Paulo Arruda

Mariana A.S. Artur

Shota Atsumi

Tamar Avin-Wittenberg

Belay Ayele

Chiara Baccolini

Christian W.B. Bachem

Andreas Bachmair

Elena Baena-Gonzalez

Luis Alonso Baez

Rajeev N. Bahuguna

Jinhe Bai

Ming-Yi Bai

Songling Bai

Yang Bai

Yuechen Bai

Margarete Baier

Thomas Baier

Soren Bak

Salma Balazadeh

Martin Balcerowicz

Pierre Baldet

Ian T. Baldwin

Raffaella Balestrini

Janneke Balk

Marilyn C. Ball

Steven G. Ball

Carlos L. Ballare

Matteo Ballottari

Edwin Jarratt Barnham

A. Cristina Barragan

Jaime Barros-Rios

Rebecca Bart

Dorothea Bartels

Laura Bartley

Gilles Basset

Diane C. Bassham

Elias Bassil

Philip David Bates

Miles Bate-Weldon

Jacqueline Batley

Henri Batoko

Alfred W. Batschauer

Raffaella Battaglia

Sébastien Baud

Isabel Baurle

Thomas Bawin

John J. Beck

Gerold J.M. Beckers

Tom Beeckman

Ludger Beerhues

Christophe Belin

Chandra Bellasio

Marian Bemer

Mohammed Bendahmane

Eva Benkova

Tom Bennett

Christoph Benning

Matthias Benoit

Andrew F. Bent

Heinrich Bente

Dominique C. Bergmann

Sara Bergonzi

Oliver Berkowitz

Mark A. Bernards

Alexandre Berr

Gerit Bethke

Michael W. Bevan

Christine A. Beveridge

Rishikesh Bhalerao

Susheel Sagar Bhat

Rahul Bhosale

Dawid Bielewicz

Brad Binder

Adam James Binns

Paul R.J. Birch

Crysten Blaby-Haas

Benjamin Blackman

Elison B. Blancaflor

Jantana Blanford

Arnold Jeffrey Bloom

Matthias Bochtler

Scott Andrew Boden

Wout A. Boerjan

Adam J. Bogdanove

Ken Boote

Justin O. Borevitz

Filipe Borges

Omar Borsani

Jan Willem Borst

Jay Bose

Miguel A. Botella

Javier Francisco Botto

Cecile Bousquet-Antonelli

John L. Bowman

Hans-Peter Braun

Tore Brembu

Eric Brenya

Phillip Brewer

Jordan R. Brock

Craig R. Brodersen

John Browse

Barry D. Bruce

Harry Brumer

Judy A. Brusslan

Qingyun Bu

Felix E. Buchert

Thomas N. Buckley

Hikmet Budak

Sean M. Bulley

Mustafa Bulut

Tessa Burch-Smith

Yogev Burko

Adrien Burlacot

Katharina Bürstenbinder

Florian A. Busch

Wolfgang Busch

Lucas Busta

Edgar B. Cahoon

Shengguan Cai

Robert Spencer Caine

Dechang Cao

Fangbin Cao

Ke Cao

Qinghe Cao

Shuqing Cao

Yangrong Cao

Matías Capella

Ana D. Caperta

Allan B. Caplan

Laurent Cappadocia

Craig H. Carlson

Andrea Carminati

Sebastien Carpentier

John P. Carr

Marc Carriquí

Micaela Carvajal

Jorge Jose Casal

Simone Diego Castellarin

Vanessa Castro-Rodriguez

Christian Danve Marco Castroverde

Paola Causin

Christopher I. Cazzonelli

Nicolas M. Cecchini

Francisco J. Cejudo

Jean-Marc Celton

Pablo D. Cerdan

Lucas A. Cernusak

Felice Cervone

Silvere Vialet Chabrand

Eunyoung Chae

Debasis Chakrabarty

Sanhita Chakraborty

Kai Xun Chan

Zhulong Chan

Hao-Xun Chang

Zhenfei Chao

Zhen-Fei Chao

Joe Chappell

Clint Chapple

Thierry Chardot

Debasis Chattopadhyay

Vishalsingh Chaudhari

Harsh Chauhan

Shu-Miaw Chaw

John M. Cheeseman

Caiyan Chen

Chen Chen

Fei Chen

Feng Chen

Gong-You Chen

Guanqun Gavin Chen

Jiang Chen

Jianghua Chen

Jiawen Chen

Jiongjiong Chen

Kunsong Chen

Ling-Ling Chen

Li-Qing Chen

Ming Chen

Mo-Xian Chen

Qian Chen

Shaojiang Chen

Tsu-Wei Chen

Wansheng Chen

Xiao-Ya Chen

Xu Chen

Xuehao Chen

Xuesen Chen

Xuewei Chen

Yinglong Chen

Yue-Qin Chen

Z. Jeffrey Chen

Zhichang Chen

Zhonghua Chen

Zhong-Hua Chen

Xiaofei Cheng

Youfa Cheng

Yunjiang Cheng

Zhihua Cheng

Christian Chevalier

Joong Hyoun Chin

Andrea Chini

Tzyy-Jen Chiou

Hye Sun Cho

Jungnam Cho

Sung-Hwan Cho

Monika Chodasiewicz

Fred W.S. Chow

Alexander Christmann

Zhizhan Chu

Kar Chun-Tan

Stephan Clemens

Hagai Cohen

Mathias Cohen

Sophie Colombié

Massiomo Confalonieri

Michael J. Considine

Lucio Conti

Natalia Correa-Aragunde

Daniel J. Cosgrove

Lorenza Dalla Costa

Sarah Courbier

Pierre-Emmanuel Courty

Jonas Coussement

Mauro Cresti

Pedro Crevillén

Haitao Cui

Yasin F. Dagdas

Jungui Dai

Xinbin Dai

Cheryl O. Dalid

Danilo M. Daloso

Vincenzo D'Amelia

Antoine Danon

Aayudh Das

Debatosh Das

Malay Das

Raju Datla

Sourav Datta

Julia M. Davies

Kevin M. Davies

Seth J. Davis

Beatrice Linda Harrison Day

Franck E. Dayan

Caroline Dean

Ross M. Deans

Seth DeBolt

Clelia De-la-Peña

Carolin Delker

Deborah J. Delmer

Néstor Fernández Del-Saz

Emilie Demarsy

Taku Demura

Fenglin Deng

Lei Deng

Wei Deng

Xian Deng

Philipp Denninger

Benjamin P. DeRidder

Guilhem J. Desbrosses

Thierry M. Desnos

Sadanand Dhekney

Christophe D'Hulst

Antonio Diaz-Espejo

Alexandra Jazz Dickinson

Nuria De Diego

Aalt D.J. van Dijk

Savithramma P. Dinesh-Kumar

Baoqing Ding

Chengqiang Ding

Fei Ding

Haidong Ding

Pingtao Ding

Xinhua Ding

Yanglin Ding

Yong Ding

Zhaojun Ding

Jose R. Dinneny

Laura Dixon

Michael Anthony Djordjevic

Susanne Dobler

Anna A. Dobritsa

Roberto Docampo

Ian C. Dodd

Gunther Doehlemann

Peter Doermann

Henrik Dohlman

Eva Dominguez

Lloyd A. Donaldson

Aiwu Dong

Hezhong Dong

Yang Dong

Zhaobin Dong

Zhicheng Dong

James Doughty

Brian P. Downes

Matthew Thomas Doyle

Georgia Drakakaki

Ingo Dreyer

Andrew Drinnan

Wolfgang Dröge-Laser

Hai Du

Huilong Du

Jiamu Du

Juan Du

Xiongming Du

Yu Du

Yujuan Du

Changqing Duan

Cheng-Guo Duan

Honglang Duan

Qiaohong Duan

Joseph G. Dubrovsky

Daniel C. Ducat

Jorge Duitama

Paul Dupree

Paula Duque

Timothy P. Durrett

Peter J. Eastmond

Alejandro A. Edera

Erika Edwards

Idan Efroni

Isabel Egea

Jürgen Eirich

Malin Elfstrand

Michael J. Emes

Motomu Endo

Timo Engelsdorf

Matthias Erb

Lara Esch

Richard V. Espley

Jose Manuel Estevez

John Richard Evans

Hiroshi Ezura

Georgina Fabro

Angela Falciatore

Jilian Fan

Liumin Fan

Longjiang Fan

Xiaorong Fan

David D. Fang

Hongcheng Fang

Jun Fang

Xiaofeng Fang

Edward E. Farmer

Jill M. Farrant

Zhangjun Fei

Kessler Felix

David A. Fell

Matyas Fendrych

Feng Feng

Jian Feng

Shouqian Feng

Xianzhong Feng

John N. Ferguson

Alisdair R. Fernie

Joerg Fettke

Ivo Feussner

Franziska Fichtner

Andreia Figueiredo

Tomas Figura

Dmitry A. Filatov

Geoffrey B. Fincher

Ruth R. Finkelstein

Iris Finkemeier

Anne-Sophie Fiorucci

Teresa B. Fitzpatrick

Jaume Flexas

Igor Florez-Sarasa

Robert Fluhr

Stefan de Folter

Eloise Foo

Joachim Forner

John E. Fowler

Christine H. Foyer

Sotirios Fragkostefanakis

Margaret Frank

Nicole Frankenberg-Dinkel

Keara A. Franklin

Peter J. Franks

Jules S. Freeman

Andrew Friend

Lorenzo Frigerio

Florian Frugier

Lucas Frungillo

Stephen C. Fry

Xiangdong Fu

Ying Fu

Anja Thoe Fuglsang

Sota Fujii

Ryo Fujimoto

Toru Fujiwara

Hidehiro Fukaki

Takeshi Fukao

Jutarou Fukazawa

Robert T. Furbank

Chihiro Furumizu

Michal Gabruk

Dominique Gagliardi

Todd A. Gaines

Gregory Alan Gambetta

Giorgio Gambino

Su-Sheng Gan

Diep R. Ganguly

Junping Gao

Xiang Gao

Gyozo Garab

Penélope Garcia-Angulo

Carlos Garcia-Mata

Liliana Elizabeth Garcia-Valencia

Mikhail Gasanov

Walter Gassmann

Christiane Gatz

Pierre Gautrat

Marina Gavilanes-Ruiz

Sonia Gazzarrini

Xianhong Ge

Xue-Jun Ge

Danny N.V. Geelen

Chris Gehring

Mary Gehring

Christoph-Martin Geilfus

Kasper van Gelderen

Stanton B. Gelvin

Andrea Genre

Jonathan I. Gent

Rene Geurts

Daniel James Gibbs

Mike Gidley

Ricardo Fabiano Hettwer Giehl

Nora Gigli-Bisceglia

Laurine M. Gilles

Stewart Gillmor

Simon Gilroy

James J. Giovannoni

Jitender Giri

Giovanni Giuliano

Francesco Giunta

Cynthia Gleason

Sean Michael Gleason

Katarzyna Glowacka

Eliezer E. Goldschmidt

Alexander Goldshmidt

Charlotte M.M. Gommers

Ben-Qiang Gong

Ji-Ming Gong

Jing Gong

Peijie Gong

Zhizhong Gong

Eliana Gonzales-Vigil

Daniel H. Gonzalez

Silvia Gonzali

Adrian Gonzalo

Daphne Goring

Xiaoping Gou

Aymeric Goyer

Emmanuelle Graciet

Murray Grant

Klaus D. Grasser

Walton Green

Christopher Grefen

Donald Grierson

Howard Griffiths

Erwin Grill

Julien Gronnier

Arthur R. Grossman

Guido Grossmann

Damian Gruszka

Marcin Grzybowski

Biao Gu

Xiaofeng Gu

Yangnan Gu

Qingmei Guan

Yajing Guan

Yuefeng Guan

Jan Günther

Hongwei Guo

Jun Guo

Li Guo

Liang Guo

Weilong Guo

Wen-Wu Guo

Woei-Jiun Guo

Ya-Long Guo

Yan Guo

Yongfeng Guo

Zhongxin Guo

Zilong Guo

Suresh Kumar Gupta

Kirstin Gutekunst

Rodrigo A. Gutiérrez

Paula Guzmán-Delgado

Takushi Hachiya

Anders Hafrén

Martin Hagemann

Candace H. Haigler

Rayko Halitschke

Yuki Hamamura

Thorsten Hamann

Olivier Hamant

Bjoern Hamberger

Fangpu Han

Guangye Han

Tianfu Han

Yuepeng Han

Kosuke Hanada

John T. Hancock

Robert D. Hancock

Tri Handoyo

Marc Hanikenne

Johannes Hanson

Mats Hansson

Guang-You Hao

Zhaodong Hao

Jeremy Harbinson

Christian S. Hardtke

Jesper Harholt

Alex Harkess

Stacey Harmer

Andrea Harper

Jeffrey F. Harper

Jake Harris

Sjon Hartman

Felix Hartmann

Thomas Hartwig

Yoko Hasegawa

Bettina Hause

Michel Havaux

Jennifer S. Hawkins

Michael J. Haydon

Junxian He

Kai He

Shengbo He

Xin-Jian He

Yan He

Yubing He

Yuqing He

Zhen He

Joshua L. Heazlewood

Antje Heese

Adrian D. Hegeman

Marcus G. Heisler

Thierry Heitz

Yrjö Helariutta

Ruediger Hell

Brent Helliker

Anja Christine Hemschemeier

Piers A. Hemsley

Amelia Henry

Isabelle Marie Henry

Raul Herrera

Diana Heuermann

Tetsuya Higashiyama

Kyoko Higuchi

Yukako Hihara

David Hildebrand

Michael Hippler

Rozenn Le Hir

Cory D. Hirsch

Joseph Hirschberg

Yuji Hiwatashi

Charlie Hodgman

Ute Hoecker

Susanne Hoffmann-Benning

Daniel Hofius

Herman Höfte

Saskia A. Hogenhout

James Holland

Andreas Holzinger

GaoJie Hong

Woo-Jong Hong

Renier A.L. van der Hoorn

Hanna Hõrak

Gorou Horiguchi

Patrick J. Horn

David P. Horvath

Michael Hothorn

Bing-kai Hou

Xingliang Hou

Yifeng Hou

Gregg A. Howe

Steen Hoyer

Maria Hrmova

Polly Yingshan Hsu

Bin Hu

Da-Gang Hu

Gui-Bing Hu

Honghong Hu

Jianping Hu

Jinyong Hu

Lingfei Hu

Xiangyang Hu

Xiuli Hu

Yilong Hu

Yuxin Hu

Jian Hua

Shuijin Hua

Ancheng Huang

Aobo Huang

Jirong Huang

Junli Huang

Kaiyao Huang

Pei-Cheng Huang

Rongfeng Huang

Shanjin Huang

Xi Huang

Xiaosan huang

Xuan Huang

Xuehui Huang

Yongyu Huang

Sariel Hübner

Ralph Hückelhoven

Elton Hudson

Pitter F. Huesgen

Jef Huisman

Yu-Hung Hung

Arthur G. Hunt

He-Qiang Huo

Enamul Huq

Abir U. Igamberdiev

Hidetoshi Iida

Takefumi Ikeda

Momoko Ikeuchi

Ryozo Imai

Takehito Inaba

Soichi Inagaki

Roger W. Innes

Massimo Iorizzo

Sandra Irmisch

Thomas Barrance Irving

Till Ischebeck

Atsushi Ishihara

Takahiro Ishikawa

Emmanuelle Issakidis-Bourguet

Hiroaki Iwai

Anjali S. Iyer-Pascuzzi

David Jackson

Peter Jahns

Mukesh Jain

Piyush Jain

Shri Mohan Jain

Manuel Jamilena

Tibor Janda

Georg Jander

Geupil Jang

Jyan-Chyun Jang

Katrin Janik

Steven Jansen

Hanna Janska

Kolby Jardine

Michael C. Jarvis

Paul Jarvis

Marianne Jaubert

Gareth Jenkins

Kaare H. Jensen

Jong-Seong Jeon

Dong-Hoon Jeong

Rae-Dong Jeong

Joseph M. Jez

Jinbu Jia

Caifu Jiang

Cai-Zhong Jiang

Danhua Jiang

Hangjin Jiang

Jiafu Jiang

Kai Jiang

Lina Jiang

Ling Jiang

Lixi Jiang

Ni Jiang

Ning Jiang

Wei Jiang

Yueming Jiang

Haruhiko Jimbo

Chong Wei Jin

Honglei Jin

Shuangxia Jin

Maofeng Jing

Arlen W. Johnson

Giles N. Johnson

Kate Mary Johnson

Kim L. Johnson

Nick Johnson

Alan M. Jones

Arthur Daniel Jones

Jonathan D.G. Jones

Matthew Alan Jones

Theodore De Jong

Magdalena M. Julkowska

Christian Jung

Ki-Hong Jung

Gerd Jürgens

Mehdi Kabbage

Pradeep Kachroo

Parvinder Kahlon

Guoyin Kai

Elias Kaiser

Tatsuo Kakimoto

Avinash C. Kamble

Nitin Uttam Kamble

Cade N. Kane

Byung-Ho Kang

Chunying Kang

Hunseung Kang

Jian Kang

Zhensheng Kang

Kostya Kanyuka

Steven D. Karlen

Rumyana Karlova

Rucha Anil Karnik

Stanislaw Karpinski

Hanns Heinz Kassemeyer

Surekha Katiyar-Agarwal

Dasmeet Kaur

Taiji Kawakatsu

Yoji Kawano

Tsutomu Kawasaki

Tomokazu Kawashima

Tesfamichael H. Kebrom

Olivier Keech

Kenneth Keegstra

Beat Keller

Markus Keller

Tim Kelliher

Pavel I. Kerchev

Stephanie C. Kerr

Rachel E. Kerwin

Felix Kessler

Sharon A. Kessler

Ahmad Khan

Nafees Khan

Inna Khozin-Goldberg

Daniel Kierzkowski

Aruna Kilaru

Beom-Gi Kim

Chul Min Kim

Hoon Kim

Hyun Uk Kim

Jeongsik Kim

Kyung Do Kim

Seungil Kim

Tetsu Kinoshita

Helmut Kirchhoff

Viktor Kirik

Yugo Kitazawa

Tamir Klein

Tatjana Kleine

Jürgen Kleine-Vehn

Marina Klemencic

Esther van der Knaap

Heather Knight

Marc R. Knight

Koichi Kobayashi

Masaru Kobayashi

Takanori Kobayashi

Yutaka Kodama

Hiroyuki Koga

Karl-Heinz Kogel

Wouter Kohlen

Bruce D. Kohorn

Takao Komatsuda

Josef Komenda

Jin Kong

Que Kong

Weiwen Kong

Zhaosheng Kong

Kai Robert Konrad

Stanislav Kopriva

Maria von Korff

Christian Körner

Dylan K. Kosma

Łucja Maria Kowalewska

László Kozma-Bognár

Ute Kraemer

Johanna Krahmer

David M. Kramer

Anja Krieger-Liszkay

Beth A. Krizek

Johannes Kromdijk

Tamar Krugmann

Vendula krynická

Jian-fei Kuang

Manoj Kumar

Barbara Kunkel

Hendrik Küpper

Miyako Kusano

Stefan Kusch

Moonhyuk Kwon

Tina Kyndt

Elia Lacchini

ZhongXiong Lai

Hon-Ming Lam

Tilman Lamparter

Amy Lanctot

John C. Larkin

Anthony William Larkum

On Sun Lau

Patrick Laufs

Thomas Laux

Amanda Lavelle

Ana M. Laxalt

Jie Le

David J. Lea-Smith

Byeong-ha Lee

Cheng-Ruei Lee

Choonseok Lee

Dong Wook Lee

Ilha Lee

Jeong Hwan Lee

Justin Lee

Myeong Min Lee

Sung Chul Lee

Yang-Seok Lee

Hendrika Leeggangers

Carolyn Lee-Parsons

Samuel Leiboff

Dario Leister

Janne Lempe

Scott Lenaghan

Ping Leng

Michael Lenhard

Jose Leon

Patricia Leon

Yehoram Leshem

Jennifer D. Lewis

Mathew Graham Lewsey

Bingbing Li

Chao Li

Chengdao Li

Chenlong Li

Chunlong Li

Dawei Li

Dawei Li

Dayong Li

En Li

Fangfang Li

Faqiang Li

Fuguang Li

Gang Li

Hong-Ju Li

Hui Li

Jia Li

Jian-Feng Li

Jianming Li

Jiejie Li

Jigang Li

Jing li

Laigeng Li

Lei Li

Man-Wah Li

Mingjun Li

Pengmin Li

Qiang Li

Qing Li

Qingshun Li

Ruixi Li

Sha Li

Shuangcheng Li

Tian hong Li

Tong Li

Weiya Li

Wen-Xue Li

Xiang-Dong Li

Xiaobo Li

Xiaolong Li

Xiaoqing Li

Xin Li

Xueyong Li

Yi Li

Yibo Li

Yuan-Yuan Li

Zheng Li

Zhengguo Li

Zhonghai Li

Ziwen Li

Gang Liang

Guohua Liang

Wanqi Liang

Wenxing Liang

Xiangxiu Liang

Yan Liang

Yu-Ya Liang

Zhikai Liang

Pan Liao

Weibiao Liao

Johannes Liesche

Mieke Van Lijsebettens

Jun Lim

Sun-Hyung Lim

Erik Limpens

Deshu Lin

Jer-Young Lin

Po-An Lin

Rongcheng Lin

Wei-Yi Lin

Wen-Hui Lin

Yao-Cheng Lin

Zongcheng Lin

Karina Van Der Linde

Hong-Qing Ling

Yu Ling

Vincenzo Lionetti

Bin Liu

Changhai Liu

Chang-Jun Liu

Dongcheng Liu

Hongtao Liu

Huanhuan Liu

Ji-Hong Liu

Kai liu

Li Liu

Mingchun Liu

Qikun Liu

Shengyi Liu

Tongkun Liu

Tzu-Yin Liu

Wende Liu

Wenxing Liu

Xigang Liu

Xingwang Liu

Yongsheng Liu

Zhenshan Liu

Zhiyong Liu

Zhongchi Liu

Zhong-Jian Liu

James R. Lloyd

Enrique Lopez-Juez

Luis Lopez-Molina

Juan Jose López-Moya

Jennifer Lorang

Francesco Loreto

Heqiang Lou

Yann-Ru Lou

Claudio Lovisolo

Rafael Lozano

Rosa Lozano-Durán

Chaofu Lu

Congming Lu

Fei Lu

Shan Lu

Shan Lu

Wang-jin Lu

Yan Lu

Yandu Lu

Yan-Li Lu

Zefu Lu

Vincenzo De Luca

Miguel de Lucas

Luigi Lucini

Jutta Ludwig-Müller

Lewis Lukens

Peter Knut Lundquist

Shiu-Cheung Lung

Chaoxi Luo

Hong Luo

Jie Luo

Xiao Luo

Christian Luschnig

Chao Ma

Chuang Ma

Fangfang Ma

Jian Feng Ma

Ligeng Ma

Pengda Ma

Wei Ma

Yi Ma

Yingxuan Ma

Yingxuan Ma

You-zhi Ma

Zhiying Ma

Ruud A. de Maagd

Peter Macheroux

David M. Mackey

Luke C.M. Mackinder

Andreas Madlung

Hiroshi Maeda

Takaki Maekawa

Massimo E. Maffei

Zoltan Magyar

Magdy M. Mahfouz

Ari Pekka Mähönen

Mateusz Majda

Pal Maliga

Hyunggon Mang

Natanael Mansilla

Marylou Mantova

Chuanzao Mao

Long Mao

Yanbo Mao

Justine Marchand

D. Blaine Marchant

Peter Marhavy

Daniel Marino

Antoine Martin

German Martinez

Jaime F. Martinez-Garcia

Alexandre Martiniere

Enrico Martinoia

Carmen Martín-Pizarro

Tatsuru Masuda

Diane Mather

Ulrike Mathesius

Sarah Mathews

Davis T. Mathieu

Yusuke Matsuda

Ryo Matsushima

Anna Matuszynska

Christophe Maurel

Scott A.M. McAdam

Conor McClune

C. Robertson McClung

Andrew G. McCubbin

Stuart McDaniel

John M. McDowell

Nate G. McDowell

Andrew J. McElrone

Heather E. McFarlane

Thomas D. McKnight

Hazel McLellan

Molly Megraw

Ewa J. Mellerowicz

Charles Melnyk

Benoit Menand

David G. Mendoza-Cozatl

Xiangzong Meng

Paz Merelo

Peter Mergaert

Christian Meyer

Blake C. Meyers

Amna Mhamdi

Todd P. Michael

Morgane Michaud

Marco Michelozzi

Daisuke Miki

Carsten Milkowski

A. Harvey Millar

Guadalupe Fernandez Milmanda

Ling Min

Akira Mine

Ray Ming

Kiril Mishev

Rowan A.C. Mitchell

Maria Mittag

Ron Mittler

Kyoko Miwa

Shin-ya Miyagishima

Junya Mizoi

Thomas Mock

Bijayalaxmi Mohanty

Torsten Möhlmann

Debra Mohnen

Antonio Molina

Ian Max Møller

Attila Molnar

Marie Monniaux

Elena Monte

Laura Moody

Sunok Moon

Steve Moose

John A. Morgan

Kentaro Mori

Miyo Terao Morita

Geoff Morris

Jenny C. Mortimer

Menachem Moshelion

Rebecca A. Mosher

Ken Motohashi

Hiroyasu Motose

Zhonglin Mou

Gregory Mouille

Steven Axel Moussu

Jozef Mravec

Gloria K. Muday

Sam T Mugford

Lena Maria Müller

Stefanie J. Müller-Schüssele

Sergi Munné-Bosch

Teun Munnik

Rana Munns

Koji Murai

Monika W. Murcha

Andrew Muroyama

James W. Murray

Jorge P. Muschietti

Angelika Mustroph

Alan M. Myers

Philippe Nacry

Miquel Nadal

Dinesh A. Nagegowda

Raimund Nagel

Thomas Nägele

Hirofumi Nakagami

Norihito Nakamichi

Yasunori Nakamura

Yuki Nakamura

Hokuto Nakayama

Toru Nakayama

Mikio Nakazono

Johnathan Napier

Satoshi Naramoto

Zeeshan Nasim

Harsh Nayyar

Andreas Nebenführ

Ladislav Nedbal

Michael M. Neff

Hilde Nelissen

Luca Nerva

Nathalie Nesi

João Abreu Neto

Matthew Neubauer

Ed Newbigin

Hieu Nguyen

Zhongfu Ni

Peter Nick

Adrienne B. Nicotra

Andreas Niebel

Thomas D. Niehaus

Erik Nielsen

Jan-Ole Niemeier

Ulo Niinemets

Selene Nikaido

Lauri Nikkanen

Basil J. Nikolau

Guogui Ning

Yuese Ning

Marc T. Nishimura

Takeshi Nishimura

Kazuhiko Nishitani

Takumi Nishiuchi

Yoshitaka Nishiyama

Ayako Nishizawa-Yokoi

Baixiao Niu

Qingfeng Niu

Krishna K. Niyogi

Steve van Nocker

Ko Noguchi

Ken-Ichi Nonomura

Gretchen B. North

Michitaka Notaguchi

Dmitri A. Nusinow

Toshihiro Obata

Yoshihisa Oda

Masaki Odahara

Mayumi Ogasa

Misato Ohtani

Kazunori Okada

Masanori Okamoto

Satoru Okamoto

Brendan M. O'Leary

Muluneh Oli

Neil E. Olszewski

Ronan O'Malley

David G. Oppenheimer

Waclaw Orczyk

Bernardo Ordas

Naomi Ori

Donald R. Ort

Oren Ostersetzer-Biran

Bo Ouyang

Esma Özhüner

Nam-Chon Paek

Ravishankar Palanivelu

Kyle Palos

Olivier Panaud

Sayantan Panda

Yongzhen Pang

Ralph Panstruga

Pramod Pantha

Florent Pantin

Madasamy Parani

Eunsook Park

Martin Parniske

Fabio Pasin

Uta Paszkowski

Sitakanta Pattanaik

Matthew J. Paul

Laurens Pauwels

Stephen Pearce

Tamara Pecenkova

Scott Charles Peck

Carsten Pedersen

Ole Pedersen

Wendy Ann Peer

Gilles Peltier

Xiongbo Peng

Jose Manuel Perez-Perez

Giorgio Perrella

Staffan Persson

Paolo Pesaresi

Reuben J. Peters

Katia Petroni

Katrin Philippar

Ronald Pierik

Miguel A. Pineros

Maria Concetta de Pinto

Joseph Chris Pires

Julien Pirrello

Séverine Planchais

John Damien Platten

Roman Pleskot

Jonathan Michael Plett

Tomas Pluskal

Yves Poirier

Maija Pollari

Jathish Ponnu

Brigitte Poppenberger

Alicia Pou

Juan Carlos del Pozo

Kalika Prasad

Salomé Prat

Brandon Pratt

Chiara Pucciariello

Holger Puchta

Sujith Puthiyaveetil

Jo Putterill

Tiancong Qi

Weiwei Qi

Xiaohua Qi

Qian Qian

Weiqiang Qian

Yong Qian

Hong Qiao

Guozheng Qin

Jinxia Qin

Rui Qin

Yonghua Qin

Yuan Qin

Yuan Qin

Jiaen Qiu

Yongjian Qiu

Zhengkun Qiu

Shenchun Qu

Leandro Quadrana

Francesca M. Quattrocchio

A.N.M. Rubaiyath Bin Rahman

Christine A. Raines

Michael T. Raissig

Prashanth Ramachandran

Vicente Ramirez

Elena Ramirez-Parra

Ashish Ranjan

Hanne N. Rasmussen

John A. Raven

G. Venugopala Reddy

Jean-Philippe Reichheld

Dugald Elgin Reid

Claire Remacle

Claire Remacle

Deyong Ren

Jose L. Reyes-Taboada

Ivan Reyna-Llorens

Adeel Riaz

Jonathan K. Richards

Andreas S. Richter

Stamatis Rigas

Christoph Ringli

Rosa M. Rivero

Thomas Roach

Bruno Robert

Stéphanie Robert

Alison W. Roberts

Jean-David Rochaix

Soizic F. Rochange

Fulton E. Rockwell

Adam B. Roddy

Kevin Rodriguez

Marianela Soledad Rodriguez

Manuel Rodriguez-Concepcion

Adrienne H.K. Roeder

Alessandra Rogato

Andrés Romanowski

Maria C. Romero-Puertas

Arnaud Ronceret

Ray J. Rose

Hernan G. Rosli

Laura Rossini

Rebecca Roston

Hatem Rouached

Sonali Roy

Yong-Ling Ruan

Francisco Rubio

Vicente Rubio

Markus Rueggeberg

Nadine Katrin Ruehr

Jose Sebastian Rufian

Nir Sade

Mayuri Sadoine

Radin Sadre

Yusuke Saijo

Atsushi Sakamoto

Yoichi Sakata

Shun Sakuma

Silvio Salvi

Jozef Samaj

Arun Sampathkumar

A. Lacey Samuels

Lacey Samuels

Diego H. Sanchez

Raquel Sánchez-Pérez

Maria-Angelica Sanclemente

Sanju A. Sanjaya

Gian Pietro Di Sansebastiano

Eriko Sasaki

Takayuki Sasaki

Christopher A. Saski

Jessica A. Savage

Tatyana Savchenko

Stefania Savoi

Samir Vishwanath Sawant

Hidetoshi Saze

Andrew P. Scafaro

Michael J. Scanlon

Anton R. Schaeffner

Ulrich Schaffrath

André Scheffel

Bernardus C.J. Schimmel

Joelle Schlapfer

Wendy M. Schluchter

Markus Schmid

Anja Schmidt

Romy R. Schmidt-Schippers

Christian Schmitz-Linneweber

Carla Schommer

M. Eric Schranz

Lukas Schreiber

Tom Schreiber

Michael Schroda

Andrea Schubert

Daniel Schubert

Marian Schubert

Margot Schulz

Karin Schumacher

Roland Ernst Schwarzenbacher

Claus Schwechheimer

Jorg Schwender

Benjamin Schwessinger

Christine Scoffoni

Steven R. Scofield

Denise Scuffi

Jose Sebastian

Mastoureh Sedaghatmehr

Ronald R. Sederoff

Maria Eugenia Segretin

Lorena Sendín

Muthappa Senthil-Kumar

Jun Sung Seo

Mitsunori Seo

Pil Joon Seo

Adél Sepsi

Roger Seymour

Sergey Shabala

Hongyan Shan

Wei Shan

Eilon Shani

John Shanklin

Xingfeng Shao

Thomas D. Sharkey

Arun K. Sharma

Rita Sharma

Sidney L. Shaw

Jie Shen

Jinbo Shen

Lisha Shen

Qian-Hua Shen

Qiufang Shen

Wenbiao Shen

Yingbai Yingbai Shen

Xianyong Sheng

Haitao Shi

Jinrui Shi

Junling Shi

Kai Shi

Suhua Shi

Yiting Shi

Toshiharu Shikanai

Mie Shimojima

Akie Shimotohno

Jay Shockey

Hagai Shohat

Allan M. Showalter

Kai Shu

Rakesh Kumar Shukla

Richard Sibout

Pierre Sicard

Jagdeep Singh Sidhu

Fernanda Silva-Alvim

Andrew J. Simkin

Carl R. Simmons

Rüdiger Simon

Thomas Sinclair

Amar Pal Singh

Keshav Singh

Shailendra Singh

Alok Krishna Sinha

Senjuti Sinharoy

João Antonio Siqueira

Robert Paul Skelton

Aleksandra Skirycz

R. Keith Slotkin

Elwira Smakowska

Ian D. Small

Geert Smant

Nicholas Smirnoff

Richard S. Smith

Dariusz Jan Smolinski

Wayne A. Snedden

Kimberley Cathryn Snowden

Kee Hoon SOHN

Kresimir Sola

Ali Soltani

David E. Somers

Fengming Song

Shun Song

Susheng Song

Weibin Song

Xiaoming Song

Uwe Sonnewald

Takashi Soyano

Edgar P. Spalding

Imogen A. Sparkes

Steven H. Spoel

Antanas Spokevicius

Nathan M. Springer

Parvathaneni Naga Srinivasu

Gary Stacey

Thomas Städler

Simon Stael

Jeffrey M. Staub

Karin Stensjö

Kathy Steppe

Eva Stoger

Sophia L. Stone

Henrik Stotz

Jens Stougaard

Josh Strable

Asa Strand

Daniela Strenkert

Anthony Studer

Nils Stührwohldt

Craig J. Sturrock

Handong Su

Tongbing Su

Senthil Subramanian

Daniela J. Sueldo

Mi Chung Suh

Na Sui

Jiaqiang Sun

Jinjing Sun

Qianwen Sun

Xiaoling Sun

Sibum Sung

Tianhu Sun

Frances Sussmilch

Takuya Suzaki

Crystal Sweetman

Aleksandra Swida-Barteczka

Ewa Swiezewska

Szymon Swiezewski

Joseph Swift

Daniel B. Szymanski

Francisco Ramón Tadeo

Goro Taguchi

Helen Tai

Teruaki Taji

Tomas Takac

Yoshinobu Takada

Taku Takahashi

Junpei Takano

Seiji Takayama

Yuri Takeda-Kimura

Daigo Takemoto

Mizuki Takenaka

Frank L.W. Takken

Michael Taliansky

Kentaro Tamura

Bao-Cai Tan

Junjie Tan

Li Tan

Lubin Tan

Shengjun Tan

Ken Tanaka

Ryouichi Tanaka

Dingzhong Tang

Guiliang Tang

Karen Kikumi Tanino

Yi Tao

Zeng Tao

Samuel H. Taylor

Thomas Teichmann

Marcella Alves Teixeira

Javier Tello

Chong Teng

Nianjun Teng

Yuanwen Teng

Ryohei Terauchi

Matthew J. Terry

Jitendra Kumar Thakur

Stephen G. Thomas

Sebastien Thomine

Bart Thomma

Beth E. Thompson

Hans Thordal-Christensen

Michael R. Thorpe

Chang-Fu Tian

Feng Tian

Jiang Tian

Zhendong Tian

Stefan Timm

Alain F. Tissier

David Tissue

lalit dev tiwari

Yuki Tobimatsu

Martina Tomasella

Alessandro Tondelli

Hongning Tong

Yiping Tong

Melis Kucukoglu Topcu

Christopher N. Topp

Masatsugu Toyota

Livio Trainotti

Prabodh Kumar Trivedi

Andrea Trotta

Marco Trujillo

Katsutoshi Tsuda

Hirokazu Tsukaya

Masayuki Tsuzuki

Viktor E. Tsyganov

Lili Tu

Matthew R. Tucker

Robert Turgeon

Ismail Turkan

Simon R. Turner

Narendra Tuteja

Stephen D. Tyerman

Akihiro Ueda

Minoru Ueda

José Manuel Ugalde

Katrin Ulrich

Peter Ulvskov

Toshihiro Umebayashi

Masaaki Umeda

Mikihisa Umehara

James G. Umen

Santosh Kumar Upadhyay

Daisuke Urano

Breeanna Urbanowicz

Uriel Urquiza

Suayib Üstün

Magalie Uyttewaal

Oswaldo Valdes-Lopez

Federico Valverde

Steffen Vanneste

Serena Varotto

Olena K. Vatamaniuk

Hans van Veen

Rafael Venado

Eloisa Vendemiatti

Muthusubramanian Venkateshwaran

Wayne K. Versaw

Lieven De Veylder

Jonathan Heras Vicente

Maïté Vicré-Gibouin

Maria Laura Vidoz

Usha Vijayraghavan

Eva Vincze

Ivana L. Viola

Kris Vissenberg

Corina Vlot

Jan de Vries

Hajime Wada

Berkley J. Walker

Cuihong Wan

Jianmin Wan

Aide Wang

Bao-sheng Wang

Bing Wang

Changquan Wang

Cun Wang

Diane Wang

Dong Wang

Dong Wang

Ertao Wang

Feifei Wang

Guannan Wang

Guanqun Wang

Guifeng Wang

Guodong Wang

Guo-Liang Wang

Haifeng Wang

Hui-Cong Wang

Jianbo Wang

Jia-Wei Wang

Kun Wang

Lei Wang

Li Wang

Lianyong Wang

Likai Wang

Lina Wang

Lirong Wang

Long Wang

Maojun Wang

Nan Wang

Nan Wang

Nian Wang

Peng Wang

Pengcheng Wang

Pengwei Wang

Ping Wang

Qiaomei Wang

Shaokui Wang

Songhu Wang

Suo-Min Wang

Tianzuo Wang

Wen-Ming Wang

We-Qing Wang

Xiangfeng Wang

Xiaofei Wang

Xiaofeng Wang

Xiaofeng Wang

Xiping Wang

Xiu-Ling Wang

Xiurong Wang

Xuemin Wang

Xuming Wang

Yanbing Wang

Yi Wang

Yiming Wang

Yingxiang Wang

Yingxiang Wang

Yizhou Wang

Yong-Fei Wang

Yu Wang

Yuanyuan Wang

Yuanyuan Wang

Yun Wang

Zhezhi Wang

Zhi-Yong Wang

Zhoufei Wang

Zonghua Wang

Pramod P. Wangikar

Claus Wasternack

Alex A.R. Webb

Chaoling Wei

Yangdou Wei

Dolf Weijers

Magdalena Weingartner

David Weiss

Daan A. Weits

Matthew Welling

Frank Wellmer

Ralf Welsch

Ruth Welti

Jing Wen

Ying-Qiang Wen

Zhifeng Wen

Bénédicte Wenden

Yiqun Weng

Yiqun Weng

Stephan Wenkel

Elizabeth Weretilnyk

Sören Werner

Eranga Wettewa

Glen Lee Wheeler

James Whelan

Steve C. Whisson

Frank F. White

Steven A. Whitham

Joshua R. Widhalm

Thomas Widiez

Emilie Wientjes

Annegret Wilde

Samuel W. Wilkinson

Neil Willey

Ben P. Williams

Ian Willick

Zoe A. Wilson

Carel W. Windt

Astrid Wingler

Jennifer H. Wisecaver

Ilka Wittig

Sebastian Wolf

Ernst J. Woltering

Hye Ryun Woo

Jesse D. Woodson

Margaret Worthington

Louwrance Peter Wright

Michael Wrzaczek

Aimin Wu

Alex Wu

Dezhi Wu

Gang Wu

Guo-Zhang Wu

Honghong Wu

Jiahe Wu

Jinhu Wu

Jun Wu

Juyou Wu

Keqiang Wu

Liang Wu

Nan Wu

Qingyu Wu

Shuang Wu

Shujing Wu

Ting Wu

Yufeng Wu

Zhiqiang Wu

Jixing Xia

Rui Xia

Tao Xia

Ye Xia

Fengning Xiang

Yong Xiang

Yun Xiang

Fangming Xiao

Guanghui Xiao

Jun Xiao

Langtao Xiao

Yang Xiaozeng

Chuanxiao xie

Deyu Xie

Fang Xie

Kabin Xie

Qiguang Xie

Qingjun Xie

Zhixin Xie

Xiu-Fang Xin

Ai-Sheng Xiong

Dongliang Xiong

Lizhong Xiong

Bin Xu

Changcheng Xu

Guohua Xu

Lin Xu

Lingfei Xu

Ming-liang Xu

Qiang Xu

Qiang Xu

Shengbao Xu

Tao Xu

Tongda Xu

Wei Xu

Yi Xu

Zheng-Yi Xu

Wei Xuan

Yuanhu Xuan

Dawei Xue

Yan Xue

Yongbiao Xue

Zheyong Xue

Iftach Yacoby

Shri Ram Yadav

Ramin Yadegari

Shaul Yalovsky

Shinjiro Yamaguchi

Naoki Yamaji

Chizuko Yamamuro

Takaki Yamauchi

Jun Yan

Shunping Yan

Wenhao Yan

Yan Yan

Zhi-Yong Yan

Marna D. Yandeau-Nelson

Bing Yang

Changqing Yang

Guang-Fu Yang

Hui Yang

Jinghua Yang

Leiyun Yang

Luming Yang

Shuhua Yang

Shu-Yi Yang

Songguang Yang

Wanneng Yang

Wenqiang Yang

Xiaoyu Yang

Xiyan Yang

Xueyong Yang

Yi Yang

Zhenyu Yang

Zhong-Bao Yang

Zhong-Nan Yang

Ziyin Yang

Gai-Fang Yao

Jia-Long Yao

Nan Yao

Wei Yao

Yuxin Yao

Jianrong Ye

Lingzhen Ye

Shenhua Ye

Wuwei Ye

Zhibiao Ye

Hsin-Hung Yeh

Kuo-Chen Yeh

Keke Yi

Hao Yin

Xiaojian Yin

Xue-ren Yin

Miingtiem Yong

Soo-Cheul Yoo

Gyeong Mee Yoon

Jinmi Yoon

Hitoshi Yoshida

Bin Yu

Deyue Yu

Diqiu Yu

Feng Yu

Hong Yu

Jianping Yu

Jingquan Yu

Shunwu Yu

Yu Yu

Li Yuan

Ling Yuan

Lixing Yuan

Yao-Wu Yuan

Yuxiang Yuan

Olga A. Zabotina

Lorenzo Zacarias

Mirko Zaffagnini

Samuel C. Zeeman

Eva van Zelm

Fanchang Zeng

Fanrong Zeng

Shaohua Zeng

Weiqing Zeng

Yunliu Zeng

Jixian Zhai

Aimin Zhang

Aying Zhang

Baocai Zhang

Cankui Zhang

Chao Zhang

Dabing Zhang

Dong Zhang

Fan Zhang

Fanfan Zhang

Feng Zhang

Jie Zhang

Jin-Song Zhang

Junhong Zhang

Liang Zhang

Liangsheng Zhang

Lixin Zhang

Meixiang Zhang

Meng Zhang

Mengxia Zhang

Mingzi Zhang

Peiqi Zhang

Qi Zhang

Ru Zhang

Rui Zhang

Tian Zhang

Tianqi Zhang

Tian-Qi zhang

Wenhua Zhang

Xiaolan Zhang

Xiuxin Zhang

Xue-Qin Zhang

Yan Zhang

Yan Zhang

Yang Zhang

Yanxia Zhang

Yijing Zhang

ying zhang

Yongjiang Zhang

Yongliang Zhang

Youjun Zhang

Yuelin Zhang

Yuyang Zhang

Zengcui Zhang

Zhengguang Zhang

Zhenhua Zhang

Zhihong Zhang

Zhonghua Zhang

Chenchen Zhao

Chunzhao Zhao

Fang-Jie Zhao

Honglong Zhao

Hongwei Zhao

Jie-Tang Zhao

Jinping Zhao

Lingxia Zhao

Meixia Zhao

Peng Zhao

Pingzhi Zhao

Yang Zhao

Yu Zhao

Yusheng Zhao

Binglian Zheng

Chengchao Zheng

Huawei Zheng

Lu Zheng

Shangwei Zhong

Ni Zhongfu

Chuanen Zhou

Chuanmiao Zhou

Hai Zhou

Junhui Zhou

Meiliang Zhou

Ming Zhou

Tao Zhou

Wenbin Zhou

Yanhong Zhou

Yihua Zhou

Yuyi Zhou

Danmeng Zhu

Hongliang Zhu

Hongyan Zhu

Qinlong Zhu

Tingting Zhu

Xiaoyang Zhu

Xinguang Zhu

Yuxian Zhu

Xiaohong Zhuang

Sabine Dagmar Zimmermann

Piotr A. Ziolkowski

Cyril Zipfel

Reimo Zoschke

Changsong Zou

Cheng Zou

## INCOMING EDITORS IN 2023

Wagner L. Arujo

Judy Brusslan

Maria Helena de Souza Goldman

Qikun Liu

Shizue Matsubara

Scott McAdam

Junpei Takano

Peng Zhang

## EDITORS PROMOTED IN 2023

Zhonghua Chen

Ronald Pierik

## OUTGOING EDITORS IN 2023

Tim Brodribb

Graham Farquhar

Alisa Huffaker

Elizabeth Kellogg

Christophe Maurel

Alex Webb

Jixian Zhai

